# Explore the mechanisms by which prenatal stress can lead to the emergence of neurodevelopmental and psychiatric disorders among children

**DOI:** 10.1192/j.eurpsy.2023.1543

**Published:** 2023-07-19

**Authors:** N. Downes

**Affiliations:** Inserm, Paris, France

## Abstract

**Introduction:**

Maternal stress experienced during pregnancy has potential lasting consequences on child development. One mechanism that can explain certain links between the activity of the maternal stress axis during pregnancy and the developmental trajectory of children is the maternal hypothalamic pituitary-adrenal (HPA) axis. Nevertheless, further exploration is needed as there are methodological limits in the existing literature, such as the lack of longitudinal data.

**Objectives:**

To fill this gap, this DEVSTRESS research project was created with the aim of increasing our understanding of the mechanisms linking prenatal maternal stress to child development using longitudinal data from the EDEN cohort study.

**Methods:**

In this sample, various bio-psycho-social data were collected: (1) maternal stress was measured during pregnancy via questionnaires assessing childhood adversity, major life events, work-related stress, anxiety, and depressive symptoms; (2) children’s emotional and behavioural problems were reported at 3, 5, 8 and 11 years, and cognitive development was assessed by psychologists at 5 years of age; (3) maternal and child hair samples provided data on the level of cortisol in the hair, which were used as a biological marker of stress and were collected at birth for both mother and child, as well as 1, 3, and 5 years after birth from children.

**Results:**

Various statistical analyses have been conducted using this data to explore the longitudinal links between self-reported maternal prenatal stress and child outcomes.

**Conclusions:**

This research project will be concluded in 2023, thus findings from the overall DEVSTRESS project and practical recommendations will be provided.

**Disclosure of Interest:**

None Declared

